# Trends and long-term variation explaining nutritional determinants of child linear growth: analysis of Bangladesh Demographic and Health Surveys 1996–2018

**DOI:** 10.1017/S1368980023002288

**Published:** 2023-12

**Authors:** Khandaker Tanveer Ahmed, Md Karimuzzaman, Sabrina Afroz, Md Moyazzem Hossain, Syeda Shahanara Huq, Faruq Abdulla, Azizur Rahman

**Affiliations:** 1 Department of Statistics, Jahangirnagar University, Savar, Dhaka 1342, Bangladesh; 2 DREXEL Dornsife School of Public Health, DREXEL University, USA; 3 School of Mathematics, Statistics and Physics, Newcastle University, Newcastle upon Tyne, Tyne and Wear, UK; 4 Cancer Care and Research Trust Bangladesh (CCRTB), Dhaka 1204, Bangladesh; 5 School of Computing and Mathematics, Charles Sturt University, Albury, NSW, Australia

**Keywords:** Malnutrition, Linear growth, Infant and child nutrition, Regression decompositions, Bangladesh

## Abstract

**Objective:**

To examine the height-for-age z-score (HAZ) of 0–35 months’ children along with stunting prevalence to identify trends, changes and available nutrition-sensitive and specific determinants that could help explain the long-term variation in child linear growth using successive Bangladesh Demographic and Health Surveys (BDHS) data from 1996 to 2018.

**Design:**

The BDHS pooled data are used for determining the key outcome variables HAZ, stunting and severe stunting. Trends, kernel-weighted local polynomial smoothing illustrations, pooled multivariable linear probability model (LPM), ordinary least squares method (OLS) and regression decomposition were used.

**Participants:**

Mothers having 0–35 months’ children, the most critical age range for growth faltering.

**Results:**

The mean HAZ increased by 0·91(±1·53) with 0·041 annual average change, while the percentages of stunting (–26·63 ± 0·54) and severe stunting (–21·12 ± 0·48) showed a reduction with 1·21 and 0·96 average annual changes, respectively. The average HAZ improvement (0·42 ± 1·56) in urban areas was less than the rural areas (1·16 ± 1·44). Similar patterns followed for stunting and severe stunting. The prenatal doctor visits (3064·65 %), birth in a medical facility (1054·32 %), breastfeeding initiation (153·18 %) and asset index (144·73 %) demonstrated a huge change. The findings of OLS, LPM and regression decomposition identified asset index, birth order, paternal and maternal education, bottle-fed, prenatal doctor visit, birth in a medical facility, vaccination, maternal BMI and ever-breastfed as influencing factors to predict the long-term changes of stunting and severe stunting.

**Conclusion:**

The nutrition-sensitive and specific factors identified through regression decomposition describing long-term variation in child linear growth should be focused further to attain the sustainable development goals.

According to the WHO, about 149 million under-five children suffer from stunting worldwide^(1–3)^, while 45 % of deaths among them are related to undernutrition^(4)^, and most of these are concentrated in low- and middle-income countries^(1,3,4)^. Asia’s stunting prevalence (52 %) is the world’s highest, and South Asian countries hold 36 % (53·7 million) of the worldwide burden of stunted children^(1,3)^. However, from 2012 to 2022, the percentage of stunted children came down from 26·3 % to 22·3 %, which was lower than WHO’s expected reduction in that period^(1)^. Low- and middle-income countries, however, face precarious malnutrition complexity, and a large number of children and women are suffering from at least one kind of malnutrition, including abnormally low weight for age (underweight), low height-for-age (stunted), low weight for height (wasted) and micronutrient deficiencies such as iron deficiency and anaemia^(5–7)^.

Stunting refers to the abnormally slow increase in children’s height or length, which results from the cumulative effects of chronic undernourishment. It represents a condition where children cannot reach their full linear growth potential. Stunted children are a particular group among those who have problems with their linear growth of height^(8)^. However, researchers have shown that early childhood stunting has negative implications, such as worse scholastic and economic attainment, as well as reduced workplace productivity^(9,10)^ It has been revealed that the physical effects of stunted children and their influence on their cognitive position contributed to poverty, particularly in low- and middle-income countries, by limiting an individual’s ability to live meaningful lives^(7)^. Stunting has functional consequences that persist into adulthood, including decreased workability or disability, and in females, increased mortality risk and adverse birth outcomes are frequently observed^(11–14)^.

Various multifaceted nutrition-sensitive and nutrition-specific factors can contribute to stunting. Nutrition-sensitive factors indirectly influence nutrition outcomes through contextual factors like socioeconomic, cultural and environmental influences. They include social determinants of health, agricultural practices, food systems and economic conditions. Nutrition-specific factors are direct interventions that directly address immediate causes of malnutrition, such as inadequate dietary intake and poor feeding practices^(15)^. Promoting breastfeeding, providing nutrient supplements and implementing nutrition education programmes may play a vital role to lessen childhood malnutrition. As with other types of malnutrition, childhood stunting is associated with the mother’s nutritional status^(16)^, for example education, wealth, age of delivery, previous birth interval and mothers’ BMI, regional and environmental factor (number of children in a household, the size of the family, improved sanitation and access to safe drinking water)^(17,18)^. Moreover, chronic illnesses associated with inadequate nutritional value, suboptimal newborns and lifetime development, and impaired health and productivity are all risk factors^(19–21)^. It is also related to proximate factors like birth order, mother’s age at marriage, immediate breastfeeding, childhood diarrhoea, vaccination status and vitamin deficiency^(22–27)^. However, particularly in Bangladesh, poverty, insufficient treatment, gender biases in decision-making, inadequate breastfeeding and complementary feeding rates, insufficient sanitation, environmental variables and recurring illnesses are only a few of the numerous factors of stunting that have been thoroughly investigated^(11,18,20,22,28,29)^. Also, many studies consider factors including the age of parents and child, health condition of both parents, birth spacing, paternal and maternal education, and access to media for determining childhood malnutrition^(7,12,13,17,20,28,30–45)^. Moreover, the study of malnutrition is exceptionally crucial for a South Asian developing country like Bangladesh, as it is a member of the world’s most stunted countries (under-five-year-old children account for 36 % of stunted children, and 12 % of these children are severely stunted) and is attempting to achieve the Sustainable Development Goals (12 of the 17 sustainable development goal contain nutritional indicators, particularly in goal 2). Additionally, Bangladesh’s nutrient indicators pattern has been ostentatiously inconsistent over time, even though the country has significantly reduced childhood stunting rates in recent decades. Conspicuously, most of the studies related to Bangladesh stunting employed a statistical measure of chi-square, or trends and time series analysis or regression models, including linear, binomial, linear, logistic or Poisson considering involve either one or two sets of Bangladesh Demographic and Health Survey (BDHS) data.

Stunting, on the other hand, is a type of malnutrition that develops before a child’s second birthday and is linked to insufficient nutritional intake due to food insecurity, poor diet quality and inappropriate prenatal nutrition, according to a recent study^(22)^. Thus, instead of studying under-five children, focusing on their early growth is more vital. As a result, we are aware of including data from children aged 0–35 months (the age range where growth faltering may occur the most^(20)^ and identify any nutrition-sensitive and specific (NSS) variables that explain the long-term variation in child linear growth).

A previous research pursued similar goals as this analysis; however, they used only the BDHS datasets from 2004 and 2017/18^(46)^. Nisbett et al. (2017) investigated Bangladesh’s nutritional change story, but their data came from various sources, including BDHS data from 1996 to 2011 and other sources^(47)^. The target population was under-five children, and mixed methods were used to investigate various aspects of nutritional deficiencies rather than focusing on NSS factors that cause children’s linear growth. Huda et al. (2018) employed the decomposition approach in a study with similar objectives to this one; however, the data were only from the BDHS between 2004 and 2014, and the target population was under-five children^(48)^.

Considering all BDHS datasets, this study aimed to investigate the long-term trends in child linear growth using data from the repeated cross-sectional BDHS survey spanning from 1996 to 2018. That is this study provides insights into the factors influencing child linear growth over time. The utilisation of this extensive dataset allows for a comprehensive examination of the long-term variation in child linear growth, contributing to our understanding of the dynamics and determinants of childhood nutritional status. A comprehensive analysis was conducted in this study, encompassing all available BDHS data from 1996 to the latest dataset in 2017–2018. This study assesses the influence of NSS determinants on child linear growth. Notably, it is worth mentioning that there has been a lack in the literature utilising these total datasets to investigate this specific topic. Furthermore, this study considered the linear growth of children aged 0–35 months using advanced statistical methods named regression decomposition, which is a novelty for studies employing BDHS data.

## Methods

### Data

The data were collected with the proper consent of the authorities from the website ‘https://dhsprogram.com/’ and they are open access for use^(49)^. Since this research was based on secondary data accumulated from the BDHS from 1996 to 2017/2018, all included mothers and infants during that time span are the study’s target population. For consistency, only mothers with children aged 0–35 months were included in the study from all the BDHS datasets. The questions included in this study were the same across the BDHS datasets from 1996 to 2017/2018. The BDHS sample includes everyone who lives in noninstitutional housing units across the country. The surveys used a sampling frame based on the Bangladesh Bureau of Statistics’ list of enumeration areas (EA) from the People’s Republic of Bangladesh’s most recent Population and Housing Census at the time of surveying. The primary sample unit for the survey is an EA, which has an average of 120 households. The country was divided into some administrative divisions, Barisal, Chittagong, Dhaka, Khulna, Mymensingh (as of 2018), Rajshahi, Rangpur (as of 2011) and Sylhet.

Each division is segmented into more zilas and upazilas. An upazila’s urban area is divided into wards, which are subdivided further into mohallas. Union parishads (UP) and mouzas within UP separate an upazila’s rural land. The survey used a stratified sample of households in two stages. In the first step, EA were chosen with a probability proportional to their size, with some in urban regions and others in rural areas. A detailed household listing operation was carried out in all the selected EA to provide a sampling frame for the second-stage selection of households. Each EA chose a set number of homes systematically to collect data. This allowed us to get accurate statistics for health and demographic indicators at national, urban, rural and divisional levels. Additionally, the data were pre-processed using the proper method, taking the aforementioned research factors into account, and then pooled according to their respective years (Table 1). However, the sampling, data collection, enumeration, design and overall description can be found in the published report of BDHS and DHS methodology^(50,51)^.

**Table 1:** List and definition of study variables

Brief title	Definition
Outcome variables	
HAZ	The height-for-age z-score (HAZ) was calculated and measured using the 2006 WHO child growth standard.
Stunting (in %)	Children’s Rate with HAZ ≤ –2 sd
Severe stunting (in %)	Children’s Rate of with HAZ ≤ –3 sd
Independent variables	
Asset index (1–10)	By using a clustered principal component analysis with five continuously measured durables, adjusted by loading weights and rescaled from 1 to 10. However, we adapted the same sort of procedure of related study^(50–52)^.
Maternal education (in years)	Mother’s Years of Schooling
Paternal education (in years)	Father’s Years of Schooling
Prenatal doctor visit (0/1)	Mothers who have done prenatal visit receive a prenatal visit by a doctor (Dummy = 1)
4^+^ ANC visits (0/1)	Mothers have been visiting four or more antenatal care (ANC) (Dummy = 1)
Iron during pregnancy (0/1)	Mothers received iron supplementation during pregnancy (Dummy = 1)
Born in medical facility (0/1)	Infant born in hospitals or in other medical facilities (Dummy = 1)
Maternal BMI (kg/m ^2^)	BMI of mother
Maternal height (0/1)	Dummy = 1, height of mother ≥ 145 cm
All vaccinations (0/1)	Dummy = 1, Bacillus Calmette-Guerin vaccine against tuberculosis; three shots of Diphtheria, Pertussis and Tetanus vaccine; two shots of Polio; measles antigen-containing vaccine between 1996 and 2014 and/or three shots of pentavalent vaccination in 2014 given to the children
Birth order (rank)	Order of the birth of a child in the family
Preceding birth interval (in years)	Interval of years between the current child’s birth and previous child
Open defecation (0/1)	Dummy = 1, household has no facilities of a latrine or a toilet
Water tube well (0/1)	Dummy = 1, tube well was the source of household drinking water
Water source-piped (0/1)	Dummy = 1, pipes were the source of household drinking water
Women’s decision-making (0/1)	Dummy = 1, Have the right of deciding how to spend her earnings
Breastfeeding duration (in months)	Months of breastfeeding to the child
Early initiation of breastfeeding (0/1)	Dummy = 1, infant breastfed within 1 h of birth.
Ever breastfed (0/1)	Dummy = 1, child has been breastfed ever
Bottle-feeding (0/1)	Dummy = 1, child was bottle-fed on the previous day

### Study variables

The height-for-age z-score (HAZ) is considered as the main dependent variable, and this research examines the prevalence of stunting and severe stunting, since stunting is now the standard measure for tracking pledges and progress toward global (and national) chronic child undernutrition targets. The HAZ is a measurement used to evaluate a child’s linear growth and nutritional status by comparing their height to the expected height for their age. Stunting is a condition characterised by a child having a significantly lower height-for-age than the norm, indicating chronic malnutrition and insufficient growth during early childhood. Severe stunting is an advanced form of stunting, signifying a more severe level of chronic malnutrition and pronounced growth faltering, with a child’s height-for-age considerably below the expected standard. Based on prior research using a similar framework and earlier regression and decomposition studies of HAZ, certain time-varying covariates at the child, family and parental levels were selected^(30,32,36,52–58)^. These covariates are simple additions to nutrition models that represent NSS determinants that are thought to affect child’s growth outcomes in children over time (Table 1)

### Statistical methods

The analysis techniques were adapted from a recent study on child stunting in Nepal^(53)^. The average and weighted prevalence of child linear growth outcome, as well as time-variant factors, were calculated for each individual survey year using ‘svyset’ command in STATA^(59)^ version 16, which accounted for the DHS sampling weight factor and adjustment for the SE for the multistage clustered sampling design have been made. This approach ensured the appropriate consideration of the complex survey design and the correlation among individuals within the same cluster^(60–62)^. The ‘anthro’ package in RStudio^(63)^ version 3·2·0 was used to calculate HAZ, with all extreme values exceeding the 6 sd range omitted^(62)^.

To determine the significant determinants of child stunting, this study used a two-stage regression and decomposition method. For the HAZ outcome, that are continuous, pooled data were used from all available BDHS to fit multivariable ordinary least squares regression models. Multivariable linear probability models (LPM) with a robust variance estimator were used to analyse the binary stunting outcomes and identify key determinants of stunting. Kernel-weighted local polynomial graphs were used to assess the linearity assumption between HAZ and continuous variables. The LPM approach was chosen for its ability to estimate associations with both continuous and binary outcomes simultaneously, allowing for a comprehensive analysis of the impact. The interpretation of estimates is based on the coefficients’ magnitude and statistical significance, indicating the change in the outcome associated with a unit change in the predictor variable. The use of LPM in similar studies supports its suitability for this analysis^(53)^.

Besides, several conventional statistical measures were also used. The following multivariate regression equation (Eq. 1) for child 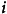 at time 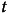 was used to assess the relation of growth outcome 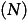 , with the 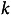 years, vectors of NSS determinants 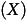 time-variable (i.e. independent variables mentioned in Table 1), vectors of time-invariant control variables 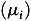 such as, maternal age (5 years interval), maternal occupation dummy variable, a child sex dummy variable, religion and division dummy variables and survey round dummy variables, dummy year variable 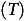 , which characterise the trend effects and SE 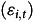 ,
(1)

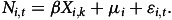




The coefficients associated with the specified determinants, displaying statistical significance with a p-value below 0·1, constitute the parameters represented by the vector of coefficients 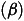 on 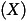 . These parameters hold a fundamental role in the investigation of factors impacting child linear growth. More precisely, the objective centres on discerning the most efficacious nutrition-sensitive and nutrition-specific determinants for elucidating variations in linear growth outcomes among children aged 0–35 months. Secondly, the parameters (estimated from the first stage regression equations) were used to perform the defined statistical decomposition (Eq. 2) (By considering the assumption of zero mean error and coefficients are time-invariant). This study analysed the earliest BDHS round in 1996 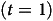 and recent BDHS round in 2018 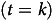 . Additionally, the analysis includes regression decomposition, calculating the mean change of each explanatory variable and multiplying the resulting coefficients on observed trends in linear growth outcomes for the 1996–2018 period to ascertain the contribution of NSS determinants (Eq. 2). This study examines the expected shift in linear growth outcomes as a function of a change in regressor during the course of the survey years (22 years), demonstrating the estimated influence of determinants and time-variant variables to child linear growth consequences.
(2)

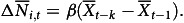




Similar comparable calculations were conducted for other NSS factors to determine, over time, how well a determinant elucidates improvements in child’s HAZ and stunting, and how well all of the independent time variables in the models illustrate changes in HAZ and stunting frequency. To ensure robustness, additional statistical analyses were performed. The comparison of LPM beta coefficients with average marginal effects from logistic regression models showed consistent findings. The Oaxaca-Blinder decomposition test revealed that 64 % of the observed coefficient differences were due to changes in covariate composition, while 36 % were attributed to changes in the coefficients themselves. Interaction terms and separate analyses for rural and urban samples provided insights into variations in predictor-child linear growth relationships. No evidence of multicollinearity was found, as variance inflation factors were below the suggested threshold of 4.

This study employed an alpha level of *P* < 0·1 as statistically significant in the regression-decomposition analysis. This choice is based on the exploratory nature of the study, aiming to identify potential associations or trends for further investigation. Previous literature has demonstrated the effectiveness of this alpha level in detecting meaningful associations in similar analyses or populations^(53)^.

## Results

### Trends in under-three child growth

**Fig. 1:**
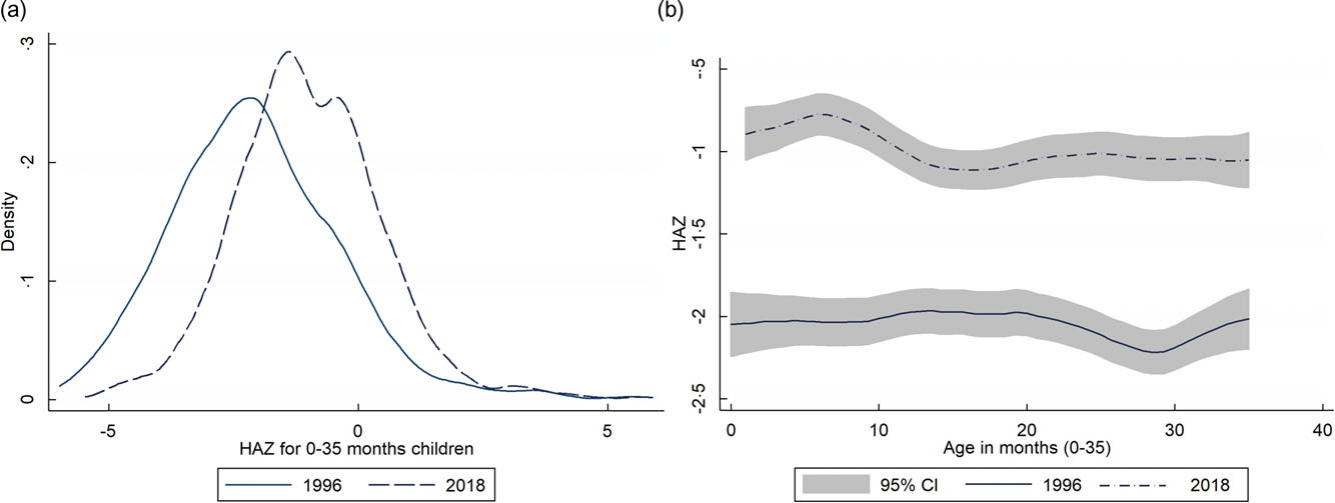
(a) Shifts in HAZ distribution among children aged 0–35 months: Kernel density estimate from BDHS 1996–2018; (b) shifts in HAZ among children aged 0–35 months by child’s age: Local polynomial smoothing predictions with 95 % CI estimated from BDHS 1996–2018.

The findings provide an overview of under three-year-old children’s HAZ, stunting and severe stunting over time, using a total sample size of 20 821 out of 35 825 mothers with children aged 0–35 months (valid samples for calculating HAZ and stunting for children 0–35 months) from seven BDHS surveys between 1996 and 2018. Bangladesh has seen a significant increase in HAZ for children aged 0–35 months, with the mean HAZ increasing by 0·91 (±1·53), while the mean HAZ increased by 1·16 (±1·44) in rural areas and by 0·42 (±1·56) in urban areas. Rural areas have a significantly lower prevalence of stunting (–33·07 ± 0·5) and extreme stunting (–23·08 ± 0·46) than urban areas (–12·71 ± 0·52) and (–12·93 ± 0·45), respectively. Between 1996 and 2018, there has been a substantial decrease in the occurrence of stunting (–26·63 ± 0·54) and severe stunting (–21·12 ± 0·48). Moreover, the mean annual reduction for HAZ, stunting and severe stunting was 0·15, 1·21 and 0·96, respectively (Table 2). Additionally, using kernel density graphs, a marginal change in the HAZ distribution was observed from 1996 to 2018 (Fig. 1). Figure 1(b) demonstrates a notable increase in the average HAZ from 1996 to 2018, while Figure 1(a) illustrates that the distribution of HAZ has remained consistent without significant changes despite this improvement.

**Table 2:** Changes in mean height-for-age z-score and stunting prevalence for different samples of children aged 0–35 months from Bangladesh Demographic and Health Surveys (BDHS) 1996–2018

	HAZ (s d)						Stunting (%) (sd)						Severe Stunting (%) (sd)					
Sample	Total	sd	Rural	sd	Urban	sd	Total	sd	Rural	sd	Urban	sd	Total	sd	Rural	sd	Urban	sd
1996	−2·05	1·71	−2·11	1·71	−1·65	1·67	56·03	0·5	57·95	0·49	44·3	0·5	30·64	0·46	31·94	0·47	22·77	0·42
2000	−1·78	1·54	−1·87	1·54	−1·54	1·51	47·26	0·5	49·39	0·5	41·36	0·49	20·65	0·40	22·01	0·41	16·86	0·37
2004	−1·72	1·44	−1·79	1·42	−1·56	1·48	44·50	0·5	46·24	0·5	40·49	0·49	17·66	0·38	18·85	0·39	14·9	0·36
2007	−1·54	1·46	−1·66	1·45	−1·33	1·44	40·03	0·49	43·60	0·5	33·93	0·47	15·42	0·36	17·1	0·38	12·55	0·33
2011	−1·48	1·70	−1·60	1·66	−1·20	1·74	40·5	0·49	42·56	0·49	36·07	0·48	18·41	0·39	19·72	0·40	15·6	0·36
2014	−1·38	1·52	−1·45	1·49	−1·25	1·55	37·87	0·49	38·88	0·49	35·92	0·48	13·14	0·34	14·01	0·35	11·47	0·32
2018	−1·14	1·46	−0·95	1·51	−1·23	1·43	29·40	0·46	24·88	0·43	31·59	0·46	9·52	0·29	8·86	0·28	9·84	0·30
Change	0·91	1·53	1·16	1·44	0·42	1·56	−26·63	0·54	−33·07	0·5	−12·71	0·52	−21·12	0·48	−23·08	0·46	−12·93	0·45
Change (%)	44·39		54·98		25·45		−47·52		−57·07		−28·69		−68·93		−72·26		−56·79	
N	20 821		12 463		8358		20 821		12 463		8358		20 821		12 463		8358	

HAZ: height-for-age z-score

Study estimates from the BDHS 1996 to 2018 rounds, using sampling weights. Stunting (%) refers to HAZ ≤ 2 sd and severe stunting (%) to HAZ ≤ 3 sd.

### Trends in nutrition-sensitive and specific determinants

With 20 821 samples, this analysis discovered major changes in certain NSS determinants. There were major increases in prenatal doctor visits (3064·65%), birth in a medical facility (1054·32%), all vaccinations (155·07%), breastfeeding initiation (153·18%), asset index (144·73%), open defecation (–96·9 %), antenatal care visits (–86·89 %) and water source: piped (65·79 %) and a minor difference in preceding birth interval (34·57%), birth order (–29·33%), breastfeeding period (–22·6%), paternal education (–20·27%), water source-tube well (–11·75%) and maternal BMI (11·01%), but this study found tiny differences in maternal education (6·75%), maternal height (5·28%) or breastfed ever (1·24%). There were no data on iron intake during pregnancy in BDHS 1996, but from BDHS 2018, it was found that 76·54% of mothers have taken iron during pregnancy (Table 3).

**Table 3:** Trends in means (sd) of NSS determinants among mothers of child aged 0–35 months

	Asset index (1–10)		Paternal education (years)		Maternal education (years)		Prenatal doctor visit (%)		ANC visits (%)		Iron during pregnancy (%)		Born in a medical facility (%)	
	Means	sd	Means	sd	Means	sd	Means	sd	Means	sd	Means	sd	Means	sd
1996	2·75	1·34	3·70	4·97	3·26	1·55	2·39	0·04	6·1	0·97	0·0		4·40	0·43
2000	3·14	1·65	3·74	6·47	3·10	1·60	27·07	0·79	0·65	0·75	33·35	0·65	10·1	0·9
2004	3·48	1·04	3·17	2·35	2·83	1·60	34·83	0·86	0·32	0·56	52·03	1·41	11·70	1·1
2007	4·11	1·27	3·54	2·24	3·29	1·50	39·46	0·91	0·35	0·66	50·16	1·17	16·5	0·94
2011	4·87	1·76	3·72	3·54	3·41	1·36	42·80	0·94	0·37	0·84	0·0		27·3	1·22
2014	5·87	1·98	3·67	2·45	3·35	1·38	39·29	0·90	0·40	1·1	0·0		23·3	0·87
2018	6·73	2·13	2·95	1·66	3·48	3·70	75·64	0·43	0·80	0·91	76·54	0·42	50·79	1·08
Change	3·98	1·57	−0·75	0·82	0·22	1·91	73·25	0·86	−5·3	0·94	76·54	0·42	46·39	0·97
Change (%)	144·73		−20·27		6·75		3064·65		−86·89		**		1054·32	
*n*	33 287		25 768		26 143		30 856		27 971		22 428		29 745	
	Preceding birth interval (years)		Open defecation (%)		Water Source: piped (%)		Water source: tube well (%)		Ever breastfed (%)		Duration of breastfeeding (months)		Maternal BMI (kg/m2)	
1996	3·76	2·00	58·1	0·62	3·8	0·8	90·2	0·8	96·6	0·3	21·99	11·75	20·07	2·32
2000	4·08	2·25	44·8	0·7	6·3	0·85	89·5	0·95	97·1	0·4	22·12	12·18	19·86	1·02
2004	3·98	2·28	41·0	0·6	7·3	0·72	84·5	0·9	98·1	0·2	23·60	12·18	19·80	0·9
2007	4·41	2·49	17·6	0·5	6·1	0·54	80·4	0·6	97·6	0·3	22·95	11·19	20·46	1·02
2011	4·52	2·49	10·8	0·4	9·0	0·6	78·3	0·67	96·3	0·4	25·40	8·86	20·87	0·9
2014	4·99	2·78	6·1	0·55	7·5	0·95	80·9	0·75	97·2	0·5	19·46	8·25	21·68	1·18
2018	5·06	2·88	1·8	0·37	6·3	0·79	79·6	0·86	97·8	0·3	17·02	15·15	22·28	3·94
Change	1·30	2·43	−56·3	0·54	2·5	0·86	−10·6	0·78	1·2	0·4	−4·97	1·6	2·21	1·12
Change (%)	34·57		−96·9		65·79		−11·75		1·24		−22·6		11·01	
*n*	26 765		35 782		35 780		35 780		35 647		35 501		35 689	
	Maternal height >= 145 cm (%)		Initiation of breastfeeding (%)		Bottle feed (%)		All vaccination (%)		Birth order (rank)					
1996	81·5	0·61	31·4	0·76	0·0		25·6	0·67	3·0	2·16				
2000	83·5	0·55	36·2	0·80	9·8	0·45	29·4	0·54	2·8	1·99				
2004	84·6	0·73	52·2	1·16	9·7	0·52	36·9	0·75	2·87	1·95				
2007	85·4	0·45	64·2	1·05	0·0		47·4	0·86	2·54	1·73				
2011	87·1	0·80	71·8	1·28	8·2	0·76	61·1	0·95	2·36	1·56				
2014	87·5	0·75	74·4	1·20	12·9	0·9	79·8	0·9	2·18	1·43				
2018	85·8	0·78	79·5	1·31	13·4	0·55	65·3	0·48	2·12	1·27				
Change	4·3	0·65	48·1	1·22	13·4	0·55	39·7	0·76	−0·88	1·07				
Change (%)	5·28		153·18		–		155·07		−29·33					
*n*	35 811		31 867		31 660		27 508		35 825					

Abbreviations: ANC: Antenatal Care;

Study estimates from the BDHS 1996 to 2018 rounds, using sampling weights.

**Change (%) of Iron during pregnancy is unavailable due to the absence of data in BDHS 1996, BDHS 2011, and BDHS 2014 surveys.

**Fig. 2:**
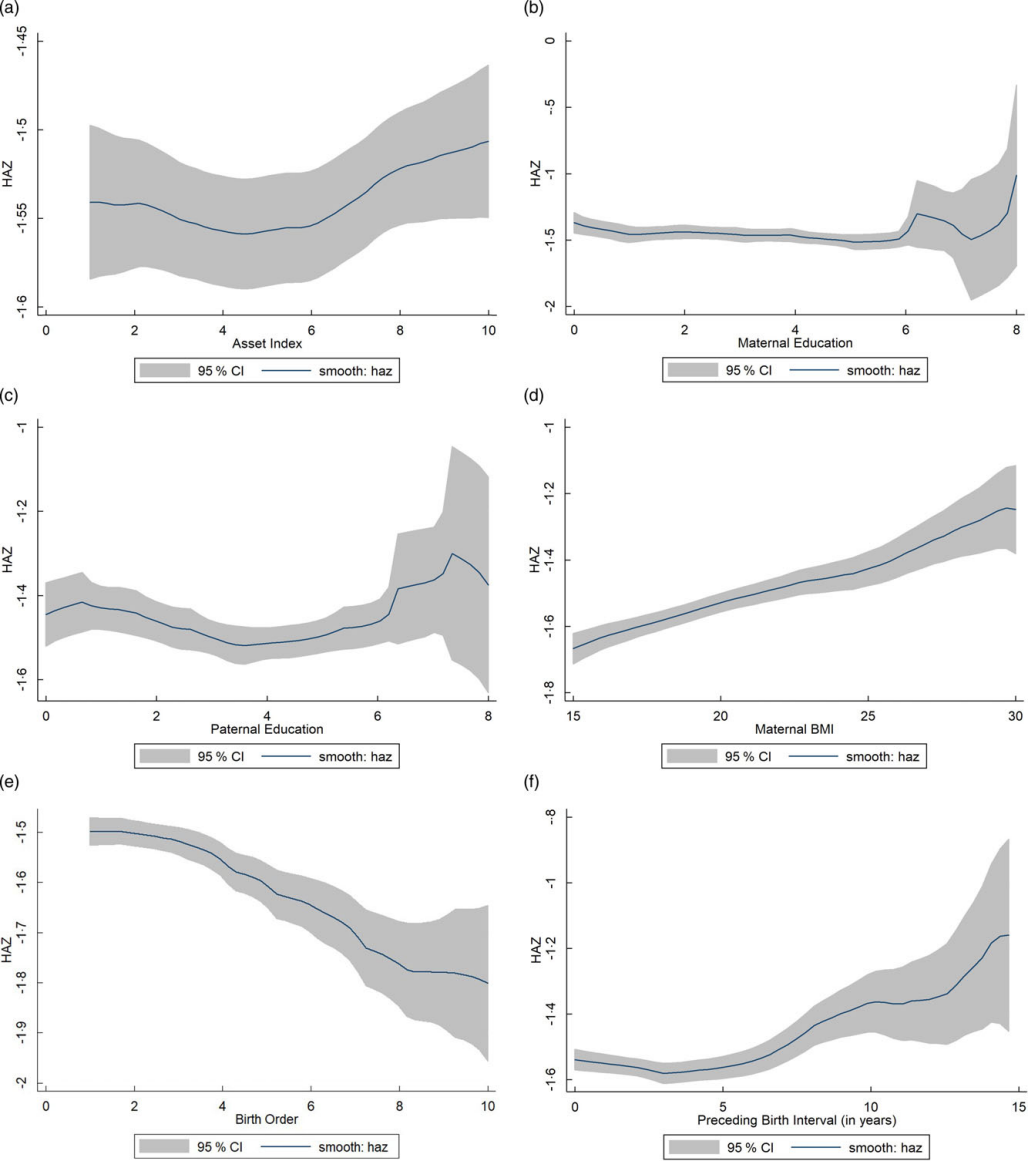
Non-parametric estimates of the relationships between height-for-age z-score (HAZ) and (a) asset index, (b) maternal education, (c) paternal education, (d) maternal BMI, (e) birth order, (f) preceding birth interval. Local polynomial smoothing predictions with 95 % CI estimated from BDHS 1996–2018.

In addition, several graphs have been shown as a non-parametric estimate to elucidate the associations amid continuous time-variant regressors and HAZ (Fig. 2). There are approximately linear relationships until a certain point; however, with the values of the continuous time-variant predictor variable being high, the linearity collapses. Moreover, the association between asset index and HAZ is almost a curve with the slope not so steep and upward with higher asset index values. Paternal and maternal education maintains an almost linear relationship with HAZ, but the linearity breaks subtly at six years of education, although paternal education with HAZ is not straight throughout. An upward linear relationship between maternal BMI and HAZ is evident from Fig. 2(d). Besides, birth order shows an approximately downward linear relation with HAZ. Lastly, the relationship between preceding birth orders and HAZ is an upward line, which is a bit curvy here and there; hence, the linearity is disrupted. To sum up, it is discernible that all the continuous time-variant independent variables have an almost linear relationship with HAZ, but when the value of these variables is high at their respective levels, due to the lower frequency of samples at that value or higher, the linearity has not seen further (Fig. 2).

### Regression decomposition

The multivariate linear regression models identified some determinants significantly associated (*P* < 0·10) with the improvement of HAZ, such as all vaccination (*β* (se): 0·002 (0·004); *P* = 0·001), paternal education (*β* (se): 0·04 (0·006); *P* = 0·07), prenatal doctor visit (*β* (se): 0·002 (0·009); *P* = 0·04), asset index (*β* (se): 0·053 (0·031); *P* = 0·06), maternal BMI (*β* (se): 0·036 (0·008); *P* = 0·04), maternal height (*β* (se): 0·017 (0·01); *P* = 0·08), initiation of breastfeeding (*β* (se): 0·003 (0·004); *P* = 0·07) and open defecation (*β* (se): –0·001 (0·002); *P* = 0·07). Although LPM run for the stunting and severe stunting estimation almost carried the same as for the determinant’s significant association like the ordinary least squares models, some exceptions are also seen, especially for maternal education (*β* (se): –0·09 (0·002); *P* = 0·06) for stunting and (*β* (se): –0·18 (0·013); *P* = 0·06) severe stunting, born in a medical facility (*β* (se): 0·002 (0·003); *P* = 0·06) for stunting and (*β* (se): 0·009 (0·003); *P* = 0·07) severe stunting, bottle feeding (*β* (se): 0·007 (0·005); *P* = 0·08) for stunting, birth order (*β* (se): –0·068 (0·01); *P* = 0·06) for severe stunting, and ever-breastfeeding (*β* (se): –0·025 (0·002); *P* = 0·009) for severe stunting (Table 4).

**Table 4:** Determinants of child (0–35 months) linear growth outcomes in pooled regression models

Dependent variable	HAZ	se	Stunting	se	Severe stunting	se
Estimator	OLS		LPM		LPM	
Asset index	0·053*	0·031	−0·15*	0·01	−0·13**	0·009
Paternal education	0·04*	0·006	−0·07*	0·01	−0·11	0·009
Maternal education	0·075	0·003	−0·09*	0·002	−0·18*	0·013
Prenatal doctor visit	0·002**	0·009	−0·004*	0·007	−0·001*	0·003
ANC visits	0·001	0·009	−0·001	0·02	−0·009	0·007
Iron during pregnancy	0·043	0·003	−0·004	0·004	−0·017	0·026
Born in a medical facility	0·006	0·005	0·002*	0·003	0·009*	0·003
Maternal BMI	0·036**	0·008	−0·021	0·011	−0·009**	0·002
Maternal height	0·017*	0·01	−0·012*	0·003	−0·025	0·026
Initiation of breastfeeding	0·003*	0·004	−0·021	0·03	0·013	0·012
Bottle feed	−0·031	0·014	0·007*	0·005	0·018	0·003
All vaccination	0·002***	0·004	−0·009	0·006	−0·013**	0·008
Birth order	0·006	0·001	−0·03	0·007	−0·068*	0·01
Preceding birth intervals	0·096	0·02	−0·013	0·04	−0·009	0·005
Open defecation	−0·001*	0·008	0·003	0·001	0·015	0·008
Water Source: piped	0·008	0·007	−0·048	0·023	−0·006	0·003
Water source: tube well	0·1	0·002	−0·032	0·011	0·09	0·008
Ever breastfed	0·07	0·006	−0·012	0·003	−0·025*	0·009
Duration of breastfeeding (months)	0·014	0·004	−0·01	0·015	−0·019	0·007
Adjusted R^2^	0·367		0·344		0·329	

HAZ: height-for-age z-score; LPM: linear probability model with a robust variance estimator; OLS: ordinary least squares.

Study estimates from the BDHS 1996 to 2018 rounds.

Stunting refers to HAZ ≤ –2 sd and severe stunting to HAZ ≤ –3 sd. The regressions include several omitted time-invariant controls, such as maternal age (5 years interval), maternal occupation dummy variable, a child sex dummy, religion and division dummy variables and survey round dummy variables.

* *P*-value < 0·05.

** *P*-value < 0·01.

*** *P*-value < 0·001.

Moreover, mean HAZ has seen gradual augmentation by years from 1996 to 2018 in 0·91 (±1·53), contributed by some nutrient determinants by a significant amount. The decomposition of those changing factors along with the asset index has been contributed most by 0·21 (23·08 %). Moreover, initiation of breastfeeding has loaded on HAZ by 0·15 (16·48 %) and prenatal doctor visit has the same contribution too, by 0·15 (16·48 %). Other factors contributed in less amount, for example all vaccination by 0·08 (8·79 %), maternal BMI by 0·08 (8·79 %), maternal height by 0·07 (7·69 %) and open defecation by 0·06 (6·59 %). Paternal education, however, contributed negatively towards HAZ by 0·03 (3·3 %). Although the mean HAZ of BDHS 2018 was improved by 0·91 than BDHS 1996, only 0·8 (87·9 %) could be estimated by the decomposition of predicted change by the determinants. Rest could be the change due to the effects of the determinants that were NS in the multivariate linear regression (Table 5).

**Table 5:** Decomposition in long-term predicted variations of regression outcomes

Dependent variable	HAZ	Stunting (%)	Severe stunting (%)
Asset index	0·21	−0·59	−0·51
Paternal education	−0·03	0·05	–
Maternal education	–	−0·02	−0·04
Prenatal doctor visit	0·15	−0·31	−0·06
Born in a medical facility	–	0·11	0·4
Maternal BMI	0·08	–	−0·02
Maternal height	0·07	−0·05	–
Initiation of breastfeeding	0·15	–	–
Bottle feed	–	0·09	–
All vaccination	0·08	–	−0·5
Birth order	–	–	0·06
Open defecation	0·06	–	–
Ever breastfed	–	–	−0·03

HAZ: height-for-age z-score.

Study estimates from the BDHS 1996 to 2018 rounds. The decomposition analysis and estimates are provided only for the independent variables that showed a significant association with HAZ, stunting and severe stunting. Stunting refers to HAZ ≤ –2 sd and severe stunting to HAZ ≤ –3 sd. The predicted nutritional change was determined using a linear decomposition at means. Changes in the mean of each time-varying variable (Table 3) were multiplied by the corresponding *β* coefficient (Table 4).

## Discussions

Bangladesh, a developing country with a large population, faces severe nutritional problems, particularly among children, and has experienced severe stunting cases over the years. However, the recent trend indicates some improvement, as this study discovered that the annual average change in height-for-age was 0·041 from 1996 to 2018. On the other hand, more significant improvement was observed in rural areas, with HAZ increasing by 0·053 annually during this period, while no such improvement was observed in urban areas, with an annual average of 0·019. The average annual change in stunting and extreme stunting was 1·21% and 0·96%, respectively. Rural areas (1·5% and 1·05 %, respectively) improved significantly more than urban areas (0·58% and 0·59%). While Bangladesh’s achievement in reducing stunting in children under three is commendable, the World Health Assembly’s and sustainable development goal programme’s target of 40% reduction in stunted children by 2025 is still a long way off^(6,64–67)^.

As demonstrated by the findings of this study, multivariate linear regression models identified several determinants significantly associated with HAZ, including factors such as all vaccination, paternal education, prenatal doctor visits, asset index, maternal BMI, maternal height, initiation of breastfeeding and open defecation. Notably, LPM used to estimate stunting and severe stunting generally aligned with the ordinary least squares models, reinforcing the significance of many determinants. However, exceptions were observed, particularly for maternal education, being born in a medical facility, bottle feeding, birth order and ever breastfeeding, where the LPM suggested potentially stronger impacts on stunting and severe stunting outcomes. These distinctions provide a nuanced understanding of the determinants influencing child linear growth and stunting, offering valuable insights into the factors affecting child health in the studied population. All of these factors have a beneficial effect on HAZ and stunting and are associated with health care. These advancements provide hope for reducing potential stunting rates. Nonetheless, some other variables, some nutrition-specific and nutrition-sensitive determinants, such as parental education, maternal height, BMI and breastfeeding, should be emphasised more to accelerate the low rate of stunting. As a developing country, Bangladesh must prioritise these points to ensure a brighter future for children and mothers. Bangladesh has tightened its grip on stunting children under five in recent years, but the rate remains around 36 %, with 12 % severely stunted. These children are at an increased risk of infection, impaired cognitive and physical development, and death. The reason for concern is that stunting’s effects may last into adulthood and may result in future reductions in these children’s work capacity^(6,11–14,33)^.

Malnutrition has a negative impact on children, their families and the entire country. Additionally, a generation’s childhood and adulthood may fail spectacularly. There have been noticeable changes in the distribution of stunting and extreme stunting as measured by endurance curves and index scales. Recent research has identified nearly identical patterns of declining recessions followed by increased socioeconomic inequality^(64,65)^. Findings revealed that stunting and severe stunting decrease significantly as the asset index increases. The asset index contributes 23·08 % to HAZ and significantly reduces stunting and extreme stunting. The association between stunting births and shorter-interval second-order births may arise due to household competition for food. Infants’ and young children’s feeding habits are critical for preventing infectious diseases and malnutrition in children. According to the WHO, infants should be breastfed exclusively for the first six months of life^(64,66,67)^. It is well-known that breastfeeding is almost universal in Bangladesh. Researchers highlighted that 43 % of children in Bangladesh are breastfed within an hour of birth, and 60 % of children receive supplementary food before reaching the sixth month^(29)^. However, this study discovered that approximately 79·5 % of children under the age of three were breastfed within an hour of birth in 2018, up from 31 % in 1996, and breastfeeding initiation has a significant HAZ effect and can account for its 16·48 % improvement. According to a Bangladesh Integrated Nutrition Program survey, 34·1 % of mothers began breastfeeding immediately after birth, while 49·9 % did so within 24 h of birth^(29)^.

Bangladesh is clearly on its way to becoming a less stunting country, as stunting and severe stunting have decreased by only 1·21 % and 0·96 % annually, respectively, despite significant improvements in prenatal doctor visits, medical facility uses, breastfeeding and all vaccinations. Surprisingly, urban areas exhibit a significantly lower rate of improvement across all three outcomes, a point that should be emphasised more. Additionally, asset index and prenatal doctor visits were identified as predictors of changes in all three outcomes, whereas paternal education and maternal height explain predicted changes in HAZ and stunting. Furthermore, maternal BMI and all infant vaccinations appear to be associated with HAZ and severe stunting. Additionally, breastfeeding for HAZ, bottle feeding for stunting and having been breastfed at any point appear to predict the changes resulting in severe stunting. Besides, maternal education and birth in a medical facility tend to be linked with stunting and severe stunting. However, based on the current rate of progress in stunting and child linear growth, the WHO and the country’s sustainable development goal programme’s target of 40 % stunting reduction by 2025 appears far from achievable^(6,64,66,67)^. On the other hand, Bangladesh will achieve its goal if widespread awareness is generated, and appropriate measures are taken to improve the circumstances of specific nutritional and nutrition-sensitive determinants (identified in the study) that contribute to HAZ and stunting.

### Strengths, limitations and future scopes

This study covers all successive data extracted from the BDHS (1996–2018) to examine trends and changes in child linear growth. Robust participant numbers, including mothers with children aged 0–35 months, contribute to a strong dataset for analysis. Statistical methods, such as kernel-weighted local polynomial smoothing, multivariable LPM, ordinary least squares and regression decomposition, are employed to explore factors associated with HAZ, stunting and severe stunting. However, there are some limitations to consider. Data collected through the BDHS may be subject to recall and reporting biases. The study’s findings are limited to the information collected in the survey and may not capture all relevant variables or nuances. Although the analysis adjusts for various factors, there may be unmeasured confounders that could influence the relationship between determinants and child linear growth. The study’s findings may be context-specific to Bangladesh and may not directly apply to other populations or countries with different socioeconomic and cultural settings. Causal relationships cannot be definitively established due to the study’s observational nature. Ecological fallacy is a potential concern as conclusions about individuals are based on group-level data. Moreover, the use of the LPM approach in this study includes potential bias from reverse causality and omitted variable bias, inability to establish causal relationships, reliance on linearity assumptions and limited interpretability for binary outcomes compared to logistic regression.

Future research can focus on several areas, including longitudinal studies to assess long-term effects, mechanistic investigations to explore underlying mechanisms, context-specific studies to identify influential factors, effectiveness evaluations of interventions, examination of socioeconomic and policy factors, exploration of parental and environmental influences, and addressing equity and disparities. Future research can also explore alternative modelling approaches, such as logistic regression or advanced machine learning techniques, to address the limitations of the LPM approach. Additionally, this study used an alpha level of *P* < 0·1 as the threshold for statistical significance in the regression-decomposition analysis. Considering more conservative approaches for statistical significance with very large datasets could be a direction for future research. These research directions can contribute to a deeper understanding of linear growth determinants and inform interventions and policies to improve child nutrition outcomes.

### Conclusion

This research tried to explore significant NSS determinants that could better explain the long-term variation in child linear growth among Bangladesh’s children aged 0–35 months, as well as to identify the condition of malnutrition through analysis and examination of stunted children. This empirical study examined fluctuations in the HAZ and the percentages of children in Bangladesh (0–35 months) from 1996 to 2018. As in stunting and severe stunting, the average annual decline is 1·21% and 0·96 %, respectively, which is satisfactory but insufficient to meet the WHO and government-set targets. However, based on endurance curves and index scales, dramatic improvements in the distribution of stunting and extreme stunting have occurred.

The asset index, on the other hand, is significantly inversely related to stunting and severe stunting. The determinants that are significantly associated with improvement in HAZ, such as universal vaccination, paternal education, prenatal doctor visits, asset index, maternal BMI, maternal height, breastfeeding initiation and open defecation, should receive increased attention to achieve more significant improvement in HAZ and stunting. Maternal education and birth in a medical facility are also two significant risk factors for stunting. A child’s birth order can also influence stunting, and the research presented here elicits that it has a significant association with stunting and severe stunting. The government of Bangladesh should place a greater emphasis on the study’s findings and underlying factors. While this analysis attempts to incorporate all possible variables, numerous additional variables need to be analysed. Shifts in attitudes over time can also be seen in next year’s BDHS data, as there have been tremendous changes in people’s attitudes over the last six years. Additionally, the longitudinal research module may evolve; further in this regard, demographic problems also play a substantial role in stunting and the development of infants.
